# Gingival Mesenchymal Stem Cells Metabolite Decreasing TRAP, NFATc1, and Sclerostin Expression in LPS-Associated Inflammatory Osteolysis *In Vivo*

**DOI:** 10.1055/s-0042-1748529

**Published:** 2022-06-21

**Authors:** Alexander Patera Nugraha, Nastiti Faradilla Ramadhani, Wibi Riawan, Igo Syaiful Ihsan, Diah Savitri Ernawati, Rini Devijanti Ridwan, Ida Bagus Narmada, Tania Saskianti, Fianza Rezkita, Andari Sarasati, Tengku Natasha Eleena Binti Tengku Ahmad Noor, Bilqis Inayatillah, Albertus Putera Nugraha, Florentina Joestandari

**Affiliations:** 1 Dental Regenerative Research Group, Faculty of Dental Medicine, Universitas Airlangga, Surabaya, Indonesia; 2 Department of Orthodontics, Faculty of Dental Medicine, Universitas Airlangga, Surabaya, Indonesia; 3 Department of Dentomaxillofacial Radiology, Faculty of Dental Medicine, Universitas Airlangga, Surabaya, Indonesia; 4 Department of Biomolecular Biochemistry, Faculty of Medicine, Universitas Brawijaya, Malang, Indonesia; 5 Stem Cell Research and Development Center, Universitas Airlangga Surabaya, Surabaya, Indonesia; 6 Department of Oral Medicine, Faculty of Dental Medicine, Universitas Airlangga, Surabaya, Indonesia; 7 Department of Oral Biology, Faculty of Dental Medicine, Universitas Airlangga, Surabaya, Indonesia; 8 Department of Pediatric Dentistry, Faculty of Dental Medicine, Universitas Airlangga, Surabaya, Indonesia; 9 Faculty of Dental Medicine, Universitas Airlangga, Surabaya, Indonesia; 10 Membership Faculty of Dental Surgery, Royal Collage of Surgery, Edinburgh University, UK; 11 Department of Basic Medical of Science, Faculty of Medicine, Universitas Airlangga, Surabaya, Indonesia; 12 Faculty of Medicine, Universitas Airlangga, Surabaya, Indonesia; 13 Faculty of Dentistry, Institute of Health Bhakti Wiyata, Kediri, Indonesia

**Keywords:** stem cells, lipopolysaccharide, medicine, infectious disease, dentistry

## Abstract

**Objective** Bone is a dynamic tissue that undergoes remodeling. During bone remodeling, there are transcription factors such as nuclear factor-activated T cells-1 (NFATc1), sclerostin, and tartrate-resistant acid phosphatase (TRAP) that are released for bone resorption. Metabolite from gingival mesenchymal stem cells (GMSCs) has the ability to activate proliferation, migration, immunomodulation, and tissue regeneration of bone cells and tissues. Furthermore, the aim of this study is to investigate the metabolite of GMSCs' effect on expression of NFATc1, TRAP, and sclerostin in calvaria bone resorption of Wistar rats.

**Materials and Methods** Twenty male healthy Wistar rats ( *Rattus norvegicus* ), 1 to 2 months old, 250 to 300 g body were divided into four groups, namely group 1 (G1): 100 µg phosphate-buffered saline day 1 to 7; group 2 (G2): 100 μg lipopolysaccharide (LPS) day 1 to 7; group 3 (G3): 100 μg LPS + 100 μg GMSCs metabolite day 1 to 7; and group 4 (G4): 100 μg GMSCs metabolite day 1 to 7. *Escherichia coli* LPS was used to induce inflammatory osteolysis on the calvaria with subcutaneous injection. GMSCs metabolite was collected after passage 4 to 5, then injected subcutaneously on the calvaria. All samples were sacrificed on the day 8 through cervical dislocation. The expression of TRAP, NFATc1, and sclerostin of osteoclast in the calvaria was observed with 1,000× magnification.

**Statistical Analysis** One-way analysis of variance and Tukey honest significant different were conducted to analyze differences between groups ( *p* < 0.05).

**Results** The administration of GMSCs metabolite can significantly decrease TRAP, NFATc1, and sclerostin expression ( *p* < 0.05) in LPS-associated inflammatory osteolysis calvaria in Wistar rats ( *R. norvegicus* ). There were significantly different TRAP, NFATc1, and sclerostin expressions between groups ( *p* < 0.05).

**Conclusion** GMSCs metabolite decrease TRAP, NFATc1, and sclerostin expression in LPS-associated osteolysis calvaria in Wistar rats ( *R. norvegicus* ) as documented immunohistochemically.

## Introduction

Bone is one of the most dynamic tissues that endure remodeling over the interaction of cellular. During bone remodeling, there is communication between osteoblasts and osteoclast at cellular level. Osteoblast has the main role in the process of bone formation, while osteoclast plays roles in the process of bone resorption. 1 Osteoclasts are differentiation of myeloid precursors that become a multinucleated cell, this happens because of consequence of supply receptor activator of cytokines nuclear factor-κB ligand (RANKL) and macrophage colony-stimulating factor (MCSF). 2 MCSF induces expression of receptor activator of NF-κB (RANK) which arouses the efficiency signaling pathway of RANK-RANKL. Some transcription factors have the responsibility to differentiate the osteoclast. The main transcription factors are nuclear factor-activated T cells c1 (NFATc1) along with NF-κB, c-Fos, and microphthalmia transcription factor. 3 4 Binding between RANK-RANKL may lead to bone resorption by inaugurating osteoclastogenesis. 5 Sclerostin or osteocyte-derived inhibitor of Wnt signaling is a tiny protein conveyed with the gene sclerostin, SOST, in osteocyte which has a crucial influence on bone remodeling that can constrain osteoblastic bone formation. 6 7 Sclerostin has the ability to compete with low-density lipoprotein receptor-related protein 5 (LRP5) and LRP6 antagonizing the pathway of canonical Wnt signaling. 8 Aside from that, during osteoclastogenesis, osteoclastic indicators such as tartrate-resistant acid phosphatase (TRAP) and sclerostin are produced, suggesting bone resorption such as in osteoporosis. According to previous research, romosozumab is a humanized monoclonal antibody to sclerostin that binds to sclerostin, allowing Wnt ligands to connect with their coreceptors, resulting in an increase in bone growth and bone mineral density. Romosozumab is a potentially new anabolic drug with a unique mode of action that has the potential to improve treatment options for osteoporosis. However, there is a limitation to the administration of romosozumab, as well as adverse effects such as arthralgia, myalgia, headache, and an increased risk of cardiovascular disease. 9 For that reason, regenerative medicine using mesenchymal stem cells (MSCs) or its metabolite may be beneficial for osteolysis treatment.

MSCs' metabolite are materials from MSC secreted products that are considered as cell-free based therapy. 10 MSCs' metabolite that consist of cell secreted has abundant benefits of therapy for several diseases such as bone defect, periodontal, arthritis, skin disease, and chronic wound. 11 12 MSCs' metabolites consist of a balanced substance between anti-inflammatory and proinflammatory which regulate the final effects. 13 MSCs secrete beneficial metabolite and exosomes, which are often just removed and become a medical waste. Nevertheless, MSCs metabolite have growth factors that provide proliferation which can affect the damaged tissue regeneration and it is beneficial to some diseases. 13 14 The reasons using MSCs for clinical application are: safe, low costs, easy to collect, less tumorigenic, and low immunogenicity. 15 16 Gingival MSCs (GMSCs) metabolite can activate proliferation, migration, immunomodulation, and tissue regeneration of the cells. 17 Previous study showed that GMSCs' metabolite that is induced by *Escherichia coli* lipopolysaccharide (LPS) in the calvaria bone of Wistar rats have the ability to diminish the number of osteoclasts and increase osteoblast number essentially. 18 LPS can stimulate proinflammatory cytokine such as tumor necrosis factor-α (TNF-α) which can affect the osteoclast formation, thus it has correlation to osteloclastogenesis. 19 However, to date, no studies have investigated the potential effect of GMSCs' metabolite on the expression of TRAP, NFATc1, and sclerostin in the osteoclast of LPS-associated inflammatory osteolysis *in vivo* . This study's hypothesis is that GMSCs' metabolite may decrease the osteoclastogenic and bone resorption-related marker such as TRAP, NFATc1, and sclerostin expressed in osteoclast in the LPS-associated inflammatory osteolysis calvaria *in vivo* . Furthermore, the aim of this study is to investigate the GMSCs' metabolite effect on the expression of TRAP, NFATc1, and sclerostin of osteoclast in the LPS-associated inflammatory osteolysis calvaria of Wistar rats ( *Rattus norvegicus* ).

## Material and Methods

### Study Design and Setting

This study protocol was approved by the ethical health committee of the Faculty of Dental Medicine, Universitas Airlangga, Surabaya, Indonesia, for animal laboratories with number 508/HRECC.FODM/VIII/2021. This study uses a true experimental laboratory with analytical posttest control group design. Lameshow's tests are used for this study to resolve the minimum sample formula; the derived total sample requirement is 20, 5 samples to every group. Blind simple random sampling technique is used for sample selection. The sample was male Wistar rats ( *R. norvegicus* ) with weights 250 to 300 g and 1 to 2 months old. All the experimental animals were healthy and had no systemic alteration condition.

### LPS-Induced Bone Resorption Animal Model

The 20 experimental animals were divided into four groups, namely group 1 (G1): 100 µg phosphate-buffered saline (PBS) day 1 to 7; group 2 (G2): 100 μg LPS day 1 to 7; group 3 (G3): 100 μg LPS + 100 μg GMSCs metabolite day 1 to 7; and group 4 (G4): 100 μg GMSCs metabolite day 1 to 7. *E. coli* LPS (LPS from *E. coli* O55:B5, EC Number: 297–473–0, Sigma Aldrich, United States) was used to induce local inflammation to activate bone resorption on the calvaria of animal model through subcutaneous injection. GMSCs' metabolite was acquired from a patent formulation owned by *Pusat Pengembangan dan Penelitian Sel Punca* (stem cell research and development center), Universitas Airlangga, Surabaya, East Java, Indonesia. GMSCs metabolite was obtained from fourth passage of cultured GMSCs with plain culture medium. The GMSCs culture medium was purified using the dialysis method to remove waste products of metabolism that were not useful, resulting in beneficial results of GMSC metabolism that contained several growth factors, chemokines, and anti-inflammatory cytokines. The dose of GMSCs' metabolite administered to the osteolysis animal model was 100 μg once a day. All samples were sacrificed on day 8 through cervical dislocation after systemic administration of rodent's anesthesia using pentobarbital 50mg/kg (pentobarbital solution, no cat: P-010, Sigma Aldrich) intraperitoneal single injection to minimize animal suffering (20–40 mL).

### Immunohistochemistry Analysis

Calvaria of the rats were cleaned with running tap water for approximately 10 to 15 minutes. After being cleaned, the calvaria were dried before washing again with 1× PBS (OneMed, Indonesia) for three times for approximately 5 to 10 minutes each time, and then again dried. After that, the sample was fixed in 10% neutral buffer formalin (Sigma Aldrich) for 4 to 7 days. Then, washed the calvaria again with 1× PBS (OneMed) three times for 5 to 10 minutes each time. To decalcify the calvaria, ethylenediaminetetraacetic acid was used for 14 to 28 days. After the calvaria have been softened, tissue processing, embedding, and sectioning were done with a microtome to obtain HPA slide. To examine the positive expression number of NFATc1, sclerostin, and TRAP in osteoclast, antibody monoclonal 1:500 dilution was used (NFATc1, Cat #MA3–024; sclerostin, Cat #MA5–23897; TRAP, Cat #MA5–12387, ThermoFisher, United States) and 3,3′-diaminobenzidine (DAB) (Abcam, United States) that is most often used in immunohistochemical (IHC) staining as a chromogen. In DAB staining, DAB is oxidized by hydrogen peroxide in a reaction typically catalyzed by horseradish peroxidase (HRP). The oxidized DAB forms a brown precipitate, at the location of the HRP, which can be visualized using light microscopy. The calculations of the positive expression number of NFATc1, sclerostin, and TRAP in osteoclasts in the LPS-associated inflammatory osteolysis calvaria were observed using an inverted light microscope (Nikon, Tokyo, Japan) with 100 × , 400 × , and 1,000× magnification in five fields of view observed by two observers. The results obtained were then recapitulated and analyzed.

### Statistical Analysis

Analysis of the data that was obtained was preceded by Kolmogorov–Smirnov test (normality test) followed by the Levene's (homogeneity test). If the data shown were normally distributed and homogeneous ( *p* > 0.05), the data were analyzed using the one-way analysis of variance test to analyze the differences between groups *p* < 0.05. The difference between each treatment group was performed by the Tukey honest significant different test *p* < 0.05. If the results were normally distributed but not homogeneous, the data were analyzed using the Kruskal–Wallis test to discover if there were differences between treatments, the difference in each treatment group was performed by the Mann–Whitney test *p* < 0.05. The Statistical Package for Social Science (SPSS) software 20.0 edition for Mac (IBM Corporation, Chicago, Illinois, United States) was used to analyze the data.

## Results

**Fig. 1:**
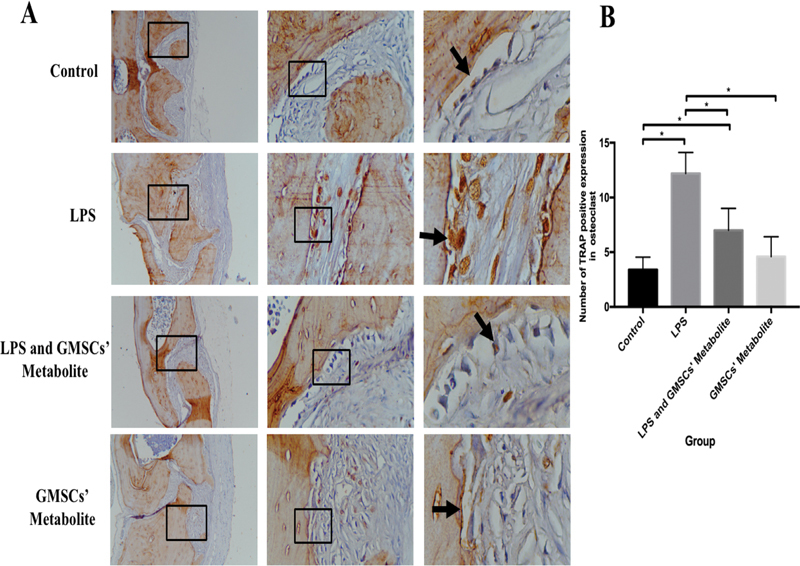
( **A** ) The (brown color) of tartrate-resistant acid phosphatase (TRAP) in osteoclasts in the calvaria in each group (black arrow) were observed by means of light inverted microscope at 100 × , 400 × , and 1,000× magnification. ( **B** ) The TRAP expression number in osteoclast in each group (* significant between groups [ *p* < 0.05]).

**Fig. 2:**
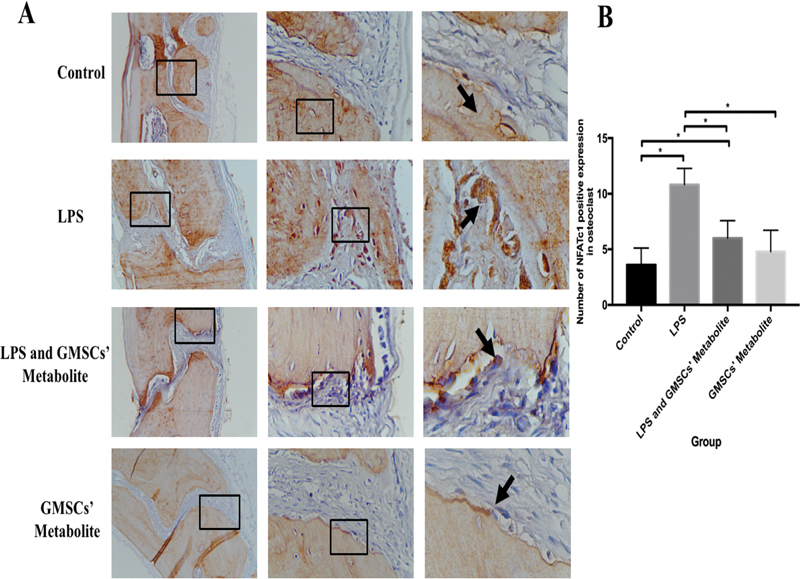
( **A** ) The positive expression (brown color) of nuclear factor-activated T cells c1 (NFATc1) in osteoclasts in the calvaria in each group (black arrow) were observed by means of light inverted microscope at 100 × , 400 × , and 1,000× magnification. ( **B** ) The NFATc1 expression number in osteoclast in each group (* significant between groups [ *p* < 0.05]).

**Fig. 3:**
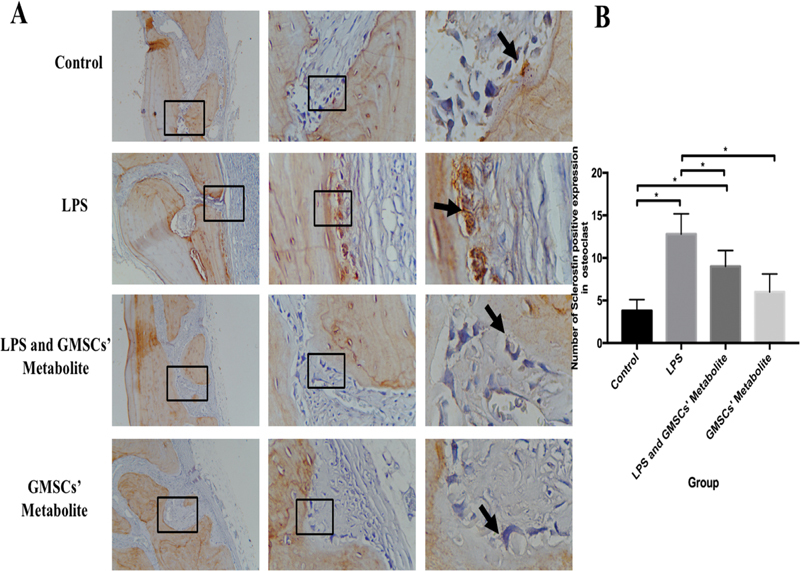
( **A** ) The (brown color) of sclerostin in osteoclasts in the calvaria in each group (black arrow) were observed by means of light inverted microscope at 100 × , 400 × , and 1,000× magnification. ( **B** ) The sclerostin expression number in osteoclast in each group (* significant between groups [ *p* < 0.05]).

In this study, data on the expression of TRAP, NFATc1, and sclerostin in the calvaria were found to be homogeneous and normally distributed ( *p* > 0.05). The positive expression (brown color) of TRAP in the osteoclast of LPS-associated inflammatory osteolysis can be seen in Fig. 1 . In addition, NFATc1 positive expression (brown color) can be seen in Fig. 2 and sclerostin positive expression (brown color) showed in Fig. 3 . The expression of TRAP, NFATc1, and sclerostin in the calvaria decreased significantly in group 3, the administration LPS combined with GMSCs' metabolite compared with the other groups ( *p* = 0.0001; *p* < 0.05). LPS injection in the calvaria increased the expression of TRAP, NFATc1, and sclerostin in the calvaria the most compared with other groups ( *p* = 0.0001; *p* < 0.05). There was no significant difference in the expression of TRAP, NFATc1, and sclerostin in the calvaria in the PBS group and GMSCs' metabolite group ( *p* = 0.0099; *p* > 0.05) ( Table 1 ).

**Table 1:** The expression of NFATc1, sclerostin and TRAP in osteoclast in the afflicted area of LPS-associated inflammatory osteolysis calvaria of Wistar rats ( *Rattus norvegicus* )

Group	NFATc1	Sclerostin	TRAP
	Mean ± SD			
PBS	3.6 ± 0.6782 ^a^	3.8 ± 0.5831 ^ab^	3.4 ± 0.5099 ^ab^
LPS	10.8 ± 0.6633 ^abc^	12.8 ± 1.068 ^acd^	12.2 ± 0.8602 ^acd^
LPS and GMSCs metabolite	6 ± 0.7071 ^b^	9 ± 0.8367 ^bc^	7 ± 0.8944 ^bc^
GMSCs metabolite	4.8 ± 0.8602 ^c^	6 ± 0.9487 ^d^	4.6 ± 0.8124 ^d^
*p* -Value	0.0053 e	0.00001 e	0.0001 e

Abbreviations: ANOVA, analysis of variance; GMSCs, gingival mesenchymal stem cells; HSD, honest significant different; LPS, lipopolysaccharide; NFATc1, nuclear factor-activated T cells c1; PBS, phosphate-buffered saline; SD, standard deviation; TRAP, tartrate-resistant acid phosphatase.

abcd Same alphabets in the same row means significant different between group examined with Tukey HSD multiple comparison test at *p* < 0.05.

e ANOVA was used with significant different at *p* < 0.05 ( *N* : 20/ *n* : 5).

## Discussion

Various conditions in the bone microenvironment could affect its homeostatic state and regulation. LPS is known to generate exaggerated osteoclastogenesis which is often found in chronic inflammatory microenvironments caused by infection. 20 GMSCs are stem cells known to possess a plethora of benefits including bone regeneration. Its metabolite as a cell-free approach is expected to exert regulatory activities to manage equilibrium of proinflammatory and anti-inflammatory activities of various bone remodeling-related cells and molecules. 21 This study demonstrated GMSCs metabolite's potential effect to regulate and reduce the inflammation caused by LPS induction that occurred in the calvaria in line with previous study result. 18

LPS is an endotoxin produced by Gram-negative bacteria that has been known to induce bone resorption through its inflammatory and osteolytic activities. 22 LPS generates its osteoclastic properties through RANKL-mediated osteoclastogenesis in osteoclast cell line, namely RAW264.7, inhibit osteoblasts formation, and reduce the mineralization rate through activating intracellular pathways. 23 24 LPS is recognized by Toll-like receptor-4 (TLR-4) and TLR-2. Thus, it is activating inflammation by stimulating various transcription factors, such as NF-κB and mitogen-activated protein kinase (MAPK). These activations would lead to secretion of TNF-α, interleukin (IL)-1, and prostaglandin E2 from osteoblasts or macrophages. 25 26 MAPK is also known to induce bone resorption through NFATc1, c-Fos, c-Jun N-terminal kinases, and extracellular signal-regulated kinases regulation, while it also alters osteoclast maturation through matrix metalloproteinase-9 (MMP-9). 27

The present study found that the GMSCs' metabolites are able to exhibit anti-inflammatory properties through various findings. NFATc1 is widely known as the master regulator of osteoclasts differentiation by regulating various osteoclastic genes, such as TRAP and cathepsin K. 3 22 NFATc1 expression was found to be substantially reduced in the LPS-induced group that was treated with GMSCs' metabolites. It was also found that the LPS-only induced group showed the highest expression of NFATc1, indicating the GMSCs' metabolite potency to prevent LPS-induced bone resorption. These results are corroborated by a previous study which stated that human gingival tissue-derived MSCs could suppress NFA1Tc expression and activation. 28 The suppression is affected by reduced TNF-α and IL-1β cells, while promoting IL-10 cells, hence promoting macrophage type 2 anti-inflammatory phenotypes and inhibit osteoclasts differentiation. 17 21

Samples in the LPS-only group showed the highest level of sclerostin, indicating that inflammation induced by LPS were activated aggressively, hence promoting bone resorption. 29 This study also found significant differences between groups, where sclerostin showed lowest expression in groups with LPS with GMSCs' metabolite. Sclerostin expression reduction would lead to increased bone formation by promoting the canonical Wnt-β catenin pathway and bone morphogenetic protein-2 gene. 30 31 Previous study found that sclerostin inhibition through utilization of antibodies altered its binding to LRP5/6, resulting in promoted bone regeneration. 30 Sclerostin was also reported to enhance RANKL expression in the Murine Osteocyte-like cell line (MLO-Y4), which is upregulated by TNF-α expression. 32 Utilization of TNF-α antagonists are reported to successfully ameliorate bone regeneration through downregulated sclerostin and RANKL expression, indicating that proinflammatory activities are necessary factors to the process. 33 GMSCs' metabolite may inhibit sclerostin expression and come from the anti-inflammatory capabilities exerted by the metabolites. Previous study elaborated that GMSCs' metabolite have distinct immunomodulatory and anti-inflammatory functions through decreasing IL-6 and TNF-α expressions, thus promoting IL-10 expression. 34

Observations on TRAP expression found that GMSCs' metabolite have the ability to inhibit osteoclasts maturation and function that lead to TRAP expression inhibition. TRAP as osteoclasts marker act in bone resorption through producing reactive oxygen species (ROS). TRAP-associated ROS are reported to break collagen and various proteins down which constitutes bone matrix. 35 TRAP expression induced by RANKL was found to be accelerated in osteocytes cocultured with sclerostin, indicating its activity in bone destruction. 36 37 GMSCs metabolite exerts its antiosteoclastic properties by inhibiting RANKL/RANK binding, leading to reduced various osteoclastic marker such as TRAP. 28 Anti-inflammatory agents are also known to inhibit various osteoclast differentiation-associated proteins, such as MMP-2, MMP-9, TRAP, cathepsin K, and NFATc1. 27 The substantial reduction of osteoclastogenesis in GMSCs' metabolite-treated groups indicate its huge potential in bone regeneration therapy. Immunomodulatory properties of GMSCs' metabolite might play the biggest part in the process to suppress inflammation. 28 GMSCs' metabolite usage is known to emphasize benefits GMSCs already possess as it has various soluble factors needed for bone regeneration, such as soluble proteins, growth factors, cytokines, and free nucleic acids. 38 Bone regeneration rate was also reported to be more robust on metabolite than MSCs-only treated group. 39 The limitations of this study was that the evaluation was performed 7 days after administration of GMSCs metabolite on the LPS-induced inflammatory osteolysis and only an IHC examination was performed.

## Conclusion

Based on the results of this study, it can be concluded that GMSCs' metabolite can inhibit TRAP, NFATc1, and sclerostin expression in LPS-induced calvaria bone resorption in Wistar rats ( *R. norvegicus* ) as documented immunohistochemically. Further research is needed to delve more about the potency of the GMSCs' metabolites to induce bone regeneration in LPS-induced bone resorption.
